# Sequencing of *hsp70* for discernment of species from the *Leishmania* (*Viannia*) *guyanensis* complex from endemic areas in Colombia

**DOI:** 10.1186/s13071-022-05438-w

**Published:** 2022-11-03

**Authors:** Juliana Hoyos, Mariana Rosales-Chilama, Cielo León, Camila González, María Adelaida Gómez

**Affiliations:** 1 Departamento de Ciencias Biológicas, Centro de Investigaciones en Microbiología Y Parasitología Tropical (CIMPAT), Universidad de los Andes, Bogota, D.C Colombia; 2 Centro Internacional de Entrenamiento E Investigaciones Médicas (CIDEIM), Campus de la Universidad Icesi (Edificio O), Cali, Colombia; 3 Universidad Icesi, Cali, Colombia; 4 Present Address: Odum School of Ecology, University of Georgia, Athens, GA 30602 USA

**Keywords:** *Leishmania* (*Viannia*), Typing, *hsp70*, Sand fly, Human, Mammalian

## Abstract

**Background:**

Colombia is ranked very high among countries with the highest numbers of endemic* Leishmania* species (*n* = 9) causing human disease*.* Although much effort has been devoted to generating simple and specific tools for *Leishmania* species identification, challenges remain in the discrimination of species belonging to the *Leishmania* (*Viannia*) *guyanensis* complex: *L.* (*V.*) *guyanensis* and *L.* (*V.*) *panamensis*.

**Methods:**

A set of seven reference strains of species belonging to the *L.* (*Leishmania*) and *L.* (*Viannia*) subgenera, clinical strains from human cases of cutaneous leishmaniasis (CL; *n* = 26) and samples collected from sylvatic mammals and sand flies (*n* = 7) from endemic areas in Colombia were analyzed in this study. The heat-shock protein 70 gene (*hsp70*) was amplified by PCR from DNA extracted from logarithmic-phase promastigotes or tissue samples, and the PCR products were sequenced. Sequence alignment was performed against a set of previously published and curated sequences, and phylogenetic analysis based on the maximum-likelihood and Bayesian inference approaches was conducted. Haplotype diversity among strains and species of the *L.* (*V.*) *guyanensis* complex was explored using a median-joining network.

**Results:**

Sequencing of the *hsp70* gene for *L.* (*Viannia*) spp. typing was comparable to species identification using isoenzyme electrophoresis or monoclonal antibodies. Complete species matching was found, except for one sylvatic sample with an identity yet unsolved. Among the *L.* (*V.*) *panamensis* clinical strains*,* two distinctive phylogenetic clusters were found to correlate with two different zymodemes: *L.* (*V.*) *panamensis* Z2.2 and Z2.3. Analysis of samples from sylvatic environments identified novel records of naturally infected wild mammal and sand fly species.

**Conclusions:**

Our results support the adequacy of *hsp70* gene sequencing as a single-locus approach for discrimination of *L.* (*Viannia*) spp., as well as for exploring the genetic diversity within the *L.* (*V.*) *guyanensis* complex.

**Graphical Abstract:**

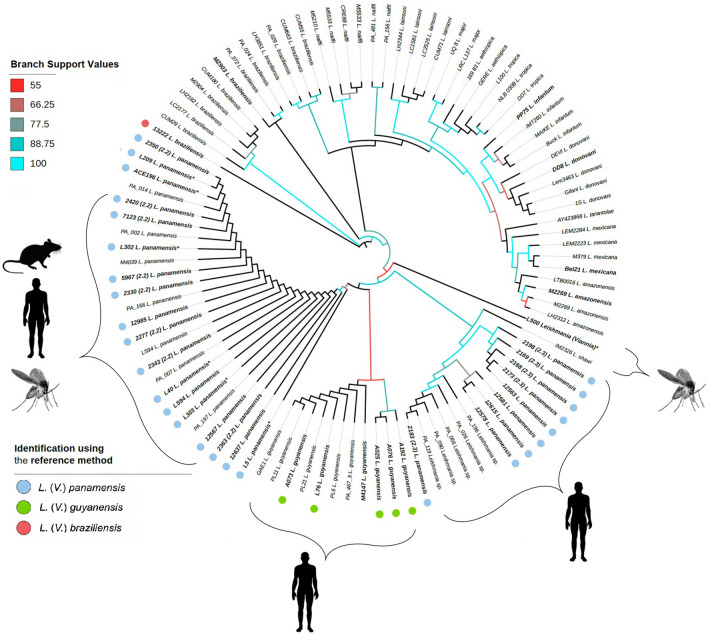

**Supplementary Information:**

The online version contains supplementary material available at 10.1186/s13071-022-05438-w.

## Background

The leishmaniases are a group of diseases caused by parasites of the genus *Leishmania*, which are transmitted through the bite of infected sand flies to humans and domestic and wild animals [1]. This spectrum of diseases is a global public health problem, with > 1.3 million new cases reported each year across 98 countries and an estimated 350 million people at risk of infection [2]. The most usual transmission cycles involve a wide, yet unspecified, range of vectors, vertebrate hosts and parasite strains, which at the same time are modulated by an uncertain number of human and environmental-related factors [3]. The leishmaniases are endemic in many areas of Central and South America [4], where usually two or more *Leishmania* species occur in sympatry [5]. Spatial co-occurrences and phylogenetic proximity of clinically relevant species pose an important challenge for species identification in cases of human infection, especially as advances in molecular biology continue to reveal a taxonomic mismatch between genetic sequence similarity and species delimitation [6].

Species belonging to the *Viannia* subgenus are the primary etiological agents of American cutaneous leishmaniasis (CL) [7]. Colombia ranks among those countries with the highest numbers of endemic *Leishmania* species (*n* = 9) causing disease in humans. These species are widely distributed across the country [8], with a predominance of *L.* (*Viannia*) *panamensis* in the north and southwest (Andean, Caribbean and Pacific regions) [8–10], causing close to 65% of all CL cases in the country, followed by *L.* (*Viannia*) *braziliensis* in the southeast (Orinoquia and Amazon regions), responsible for 30% of annual cases. Infections with *L.* (*Viannia*) *guyanensis* are less frequent (approx. 1% annually), with the exception of critical outbreaks, such as the 2003–2004 outbreak in Chaparral, Tolima, which affected > 2000 people [11]. The eco-epidemiological dynamics of some of these species has changed over time from sylvatic to domestic cycles [12, 13], a development which favors encounters with new ecological and biological pressures and results in diversification of the species distribution throughout the territory of the country.

The importance of trustworthy species typing in *Leishmania* (*Viannia*) human infections arises from its potential relevance in clinical practice. This is illustrated by: (i) mucosal disease being more frequently associated with *L.* (*V.*) *braziliensis* infections [8]; (ii) the lower susceptibility of *L.* (*V.*) *braziliensis* to miltefosine compared to *L*. (*V.*) *panamensis* or *L.* (*V.*) *guyanensis* [14]; and (iii) *L.* (*V.*) *guyanensis* infections being more likely to be completely resolved after antimonial treatment compared to infections caused by *L.* (*V.*) *braziliensis* and *L.* (*V.*) *panamensis* [15]. However, it is also true that phenotypic diversity within a species occurs, as demonstrated by the range of clinical and therapeutic outcomes associated with *L.* (*V.*) *panamensis* or *L.* (*V.*) *braziliensis* infections [16–18].

During the last three decades, multilocus enzyme electrophoresis (MLEE) has been used as the gold standard for the identification of *Leishmania* species [19]. This technique has proven reliable for defining interspecific boundaries in *Leishmania*, as well as intraspecific variations, such as those found in strains of the *L.*(*V.*) *guyanensis* and *L.* (*V.*) *braziliensis* complexes, by constraining specific populations based on their isoenzyme profile (zymodeme) [20, 21]. The *L.*(*V.) guyanensis* complex constitutes a monophyletic complex in which *L.* (*V.*) *panamensis* and *L.* (*V.*) *guyanensis* species assemble in two sub-clusters. However, phenetic and phylogenetic analyses performed on MLEE and random amplified polymorphic DNA (RAPD) data [20, 22] have not shown strict boundaries between these species, introducing a challenge for molecular-based typing.

Assessment of *Leishmania* species diversity in Colombia has focused on human host isolates, as studies in naturally infected vectors and vertebrates from endemic or sylvatic areas are scarce [23]. This is primarily due to the difficulty in capturing and isolating parasites from infected vectors or mammalian reservoirs due to low infection rates [24]. Therefore, tools that allow *Leishmania* typing directly from either clinical or biological (vector and reservoir) samples are urgently needed. The aim of the study reported here was to evaluate the performance of heat-shock protein 70 gene (*hsp*70) sequencing as an alternative for *Leishmania* species genotyping of strains isolated from cases of human CL in Colombia, and to provide proof-of-concept of its utility in species typing from primary tissue samples from sylvatic mammals and sand flies collected in areas of Colombia with high endemicity of CL.

## Methods

### *Leishmania* reference strains

Seven *Leishmania* reference strains obtained from the CIDEIM BioBank were used to confirm the accuracy of the typing protocol: *Leishmania* (*V.*) *panamensis* (MHOM/PA/71/LS94), *Leishmania* (*V.*) *guyanensis* (MHOM/BR/75/M4147), *Leishmania* (*V.*) *braziliensis* (MHOM/BR/75/M2903), *Leishmania* (*L.) infantum* (MHOM/BR/74/PP75), *Leishmania* (*L.*) *amazonensis* (MHOM/BR/73/M2269), *Leishmania* (*L.*) *mexicana* (MHOM/BZ/82/BEL21), *Leishmania* (*L.*) *donovani* (MHOM/IN/80/DD8) and *Leishmania* (*V.*) *naiifi* (MDAS/BR/79/M5533). Promastigotes were maintained at 25 °C in complete RPMI medium (supplemented with 10% heat-inactivated fetal bovine serum [Gibco™, Thermo Fisher Scientific, Waltham, MA, USA], 1% glutamine, 100 U/ml penicillin and 100 μg/mL streptomycin). Logarithmic-phase promastigotes were harvested by centrifugation, washed in phosphate-buffered saline and solubilized in lysis buffer for DNA extraction.

### Selection of clinical strains

Clinical strains isolated from 26 patients with CL were obtained from the CIDEIM BioBank. All strains had been previously typed as *L.* (*V.*) *panamensis* or *L*. (*V.*) *guyanensis* with either monoclonal antibodies or by isoenzyme electrophoresis. Additional details for these strains, including collection sites and MLEE or antibody species typing, are described in Table 1.

**Table 1:** Features of the clinical samples included in this study

Strain code	Geographical origin within Colombia (department)	Source	Species identity using reference typing method	Reference typing method	Year of isolation	Species typing based on *hsp70* sequencing	*Leishmania hsp70* sequence accession number
12581	Valle del Cauca	Human	*L.* (*V.*) *panamensis*	Monoclonal antibodies and MLEE	2016	*L.* (*V.*) *panamensis*	ON885965
12615	Valle del Cauca	Human	*L.* (*V.*) *panamensis*	Monoclonal antibodies	2016	*L.* (*V.*) *panamensis*	ON806897
12578	Valle del Cauca	Human	*L.* (*V.*) *panamensis*	Monoclonal antibodies	2016	*L.* (*V.*) *panamensis*	ON806896
2183	Nariño	Human	*L.* (*V.*) *panamensis*	Monoclonal antibodies and MLEE	1985	*L.* (*V.*) *panamensis*	ON806884
12563	Valle del Cauca	Human	*L.* (*V.*) *panamensis*	Monoclonal antibodies	2016	*L.* (*V.*) *panamensis*	ON806894
2198	Nariño	Human	*L.* (*V.*) *panamensis*	Monoclonal antibodies and MLEE	1984	*L.* (*V.*) *panamensis*	ON806885
2168	Nariño	Human	*L.* (*V.*) *panamensis*	Monoclonal antibodies and MLEE	1984	*L.* (*V.*) *panamensis*	ON806881
2173	Nariño	Human	*L.* (*V.*) *panamensis*	Monoclonal antibodies and MLEE	1984	*L.* (*V.*) *panamensis*	ON806883
2169	Nariño	Human	*L.* (*V.*) *panamensis*	Monoclonal antibodies and MLEE	1984	*L.* (*V.*) *panamensis*	ON806882
2363	Nariño	Human	*L.* (*V.*) *panamensis*	Monoclonal antibodies and MLEE	1984	*L.* (*V.*) *panamensis*	ON806890
5967	Risaralda	Human	*L.* (*V.*) *panamensis*	Monoclonal antibodies and MLEE	2011	*L.* (*V.*) *panamensis*	ON806892
2277	Nariño	Human	*L.* (*V.*) *panamensis*	Monoclonal antibodies and MLEE	1985	*L.* (*V.*) *panamensis*	ON806886
7123	Valle del Cauca	Human	*L.* (*V.*) *panamensis*	Monoclonal antibodies and MLEE	2012	*L.* (*V.*) *panamensis*	ON806893
2420	Nariño	Human	*L.* (*V.*) *panamensis*	Monoclonal antibodies and MLEE	1985	*L.* (*V.*) *panamensis*	ON806891
2343	Nariño	Human	*L.* (*V.*) *panamensis*	Monoclonal antibodies and MLEE	1985	*L.* (*V.*) *panamensis*	ON806888
2330	Nariño	Human	*L.* (*V.*) *panamensis*	Monoclonal antibodies and MLEE	1985	*L.* (*V.*) *panamensis*	ON806887
12567	Valle del Cauca	Human	*L.* (*V.*) *panamensis*	Monoclonal antibodies	2016	*L.* (*V.*) *panamensis*	ON806895
2350	Nariño	Human	*L.* (*V.*) *panamensis*	Monoclonal antibodies and MLEE	1985	*L.* (*V.*) *panamensis*	ON806889
12,985	Valle del Cauca	Human	*L.* (*V.*) *panamensis*	Monoclonal antibodies	2017	*L.* (*V.*) *panamensis*	ON885966
12,637	Valle del Cauca	Human	*L.* (*V.*) *panamensis*	Monoclonal antibodies	2016	*L.* (*V.*) *panamensis*	ON806898
A076	Tolima	Human	*L.* (*V.*) *guyanensis*	Monoclonal antibodies	2006	*L.* (*V.*) *guyanenesis*	ON806900
A073	Tolima	Human	*L.* (*V.*) *guyanensis*	Monoclonal antibodies	2006	*L.* (*V.*) *guyanenesis*	ON806899
A192	Tolima	Human	*L.* (*V.*) *guyanensis*	Monoclonal antibodies	2009	*L.* (*V.*) *guyanenesis*	ON806901
A025	Tolima	Human	*L.* (*V.*) *guyanensis*	Monoclonal antibodies	2006	*L.* (*V.*) *guyanenesis*	ON885964
L76	Amazonas	Human	*L.* (*V.*) *guyanensis*	Monoclonal antibodies	1982	*L.* (*V.*) *guyanenesis*	ON885963
13222	Valle del Cauca	Human	*L.* (*V.*) *braziliensis*	Monoclonal antibodies	2018	*L.* (*V.*) *braziliensis*	ON548520

*hsp70* Heat-shock protein 70 gene,*L. (V.)* *Leishmania* (*Viannia*),* MLEE* Multilocus enzyme electrophoresis

### Sand flies and wild mammals

*Leishmania* DNA was obtained from samples previously collected and stored at the Research Center for Microbiology and Tropical Parasitology (CIMPAT) at the Universidad de los Andes (Uniandes). Five pools (*n* = 20 specimens per pool) of *Lutzomyia gomezi* from Córdoba department collected in 2016 [25], one pool of *Psychodopygus panamensis* from Buenaventura collected in 2019 and a tissue DNA sample of one sylvatic rodent, *Oecomys* sp., from San Joaquin, Cundinamarca collected in 2019 were included (see Table 2 for sampling sites). *Leishmania* detection in these samples was previously achieved by PCR analysis of kinetoplast DNA (kDNA) for the *P. panamensis* [26] pool and by analysis of the internal transcribed spacer 1 (ITS1) for the *L. gomezi* and *Oecomys* sp. samples [27].

**Table 2:** Source of vector and mammalian samples

Sample code	Location	Vector/host species	*Leishmania* detection method	Collection year	*Leishmania hsp70* sequence accession number
L500	Valle del Cauca	*Psychodopygus panamensis*	kDNA-PCR	2019	ON806906
L5	Cordoba	*Lutzomyia gomezi*	ITS1	2016	ON806903
L302	Cordoba	*Lutzomyia gomezi*	ITS1	2016	ON885962
L209	Cordoba	*Lutzomyia gomezi*	ITS1	2016	ON806905
L303	Cordoba	*Lutzomyia gomezi*	ITS1	2016	ON885961
L40	Cordoba	*Lutzomyia gomezi*	ITS1	2016	ON806904
ACE196	Cundinamarca	*Oecomys* sp.	ITS1	2019	ON806902

*ITS1* Internal transcribed spacer 1, *kDNA* kinetoplast DNA

### DNA extraction, PCR amplification of *hsp70* gene and amplicon purification

Genomic DNA was isolated with the DNeasy extraction kit (Qiagen, Hilden, Germany) according to the manufacturer's protocol. Next, 2.5 μl of extracted DNA was used in PCR reactions performed in a final reaction volume of 25 μl, following the typing protocol and sequencing algorithm designed by [28], with the PCR parameters adapted for the used of Platinum® Taq DNA Polymerase High Fidelity (Life Technologies™, Thermo Fisher Scientific). We used the F25 and R1310 primers to amplify a 1286-bp fragment of the *hsp*70 gene (PCR-F). Samples for which no amplification product was detected were re-tested with two additional PCRs: (i) PCR-N (500-bp fragment), using primers F25 and R617; and (ii) PCR-T (700-bp fragment), using primers 6F and R1310. The amplified products were analyzed by electrophoresis in Sybr Safe-stained 2% agarose gels, the band size was confirmed and PCR products were purified using the QIAquick Gel Extraction Kit (Qiagen) following the manufacturer's protocol.

### Sequence editing and alignment

Bidirectional sequencing of the amplification products was conducted by Macrogen Genomics Laboratories (Macrogen Inc., Seoul, South Korea) using 4F and 6R primers [28]. The resulting sequences were edited and aligned in the DNA STAR program (DNASTAR, Inc., Madison, WI, USA). We performed a search of *Leishmania* sequences in GenBank using the Basic Local Alignment Search Tool (BLAST) to locate additional related strains. A first alignment was made with the sequences generated in this study alongside 59 reference sequences (Additional file 1: Table S1) retrieved from NCBI (https://www.ncbi.nlm.nih.gov) [29–35]. All sequences were aligned and edited using Muscle implemented in MegAlign Pro within (DNASTAR, Inc.). For haplotype diversity analysis, a second alignment was constructed that included only sequences of *L. *(*V.*) *panamensis*.

### Phylogenetic analysis

Phylogenies were inferred using maximum likelihood (ML) and Bayesian inference (BI). The ML tree was built using IQ-TREE [36]. The best-fitting model of molecular evolution for the ML tree was selected based on the Bayesian information criterion (BIC) using the ModelFinder command in IQ-TREE. To assess branch support, the IQ-TREE analyses used the ultrafast bootstrap approximation (UFboot) with 1000 replicates and the SH-like approximate likelihood ratio test (SH-aLRT) also with 1000 bootstrap replicates [37]. For the BI-inferred tree, we used the program PartitionFinder v2.1.1 [38] and Jmodeltest to select the most appropriate substitution model. Bayesian analyses were carried out using the program MrBayes v3.2 [39]. Two parallel sets of four simultaneous Monte Carlo Markov chains (3 hot and 1 cold) were run for 10,000,000 generations, and the trees were sampled for every 1000 generations. Temperature burn-in was set to 25% (burn-in frac = 0.25). To speed up convergence, we employed the ML tree as the starting value (‘starting tree’) for the tree parameter (tau) and the branch length parameter (V) with the MrBayes v3.2 commands: ‘startvals tau = mystarttree V = mystarttree.’ The maximum clade credibility tree (MCC) was displayed and edited in the online tool ITOL.

### Test of monophyly

Bayes factor (BF) comparisons of constrained and unconstrained tree topologies were used to test for monophyly of the *L.* (*V.*) *panamensis* strains belonging to the zymodemes 2.2 and 2.3 based on the *hsp70* sequencing. To do this, we compared an unconstrained hypothesis (no changes at all) with a constrained hypothesis in which all *L.* (*V.*) *panamensis* zymodemes were constrained to monophyly. We used MrBayes to calculate the harmonic mean estimator of marginal likelihood. BFs were calculated as the difference of harmonic mean estimators of the two models (constrained vs unconstrained) in log units; a log difference of 3–5 is considered to be strong evidence, and a difference > 5 is considered to be very strong evidence in favor of the better model [40]. BI of constrained topologies was run with the same settings as described above for unconstrained phylogenetic analysis.

### Genetic diversity

To quantify diversity among co-occurring *L.* (*V.*) *panamensis* strains in each department, 31 sequences (25 from clinical isolates, 5 from sand flies and 1 from a rodent) plus *L.* (*V.*) *panamensis* strains from Miranda et al. [41] were analyzed to search for polymorphisms using the DnaSP 6.10.03 software package. Gaps/missing bases were not considered, and invariable sites were removed. Genetic diversity was described by estimating the number of segregating sites (S), nucleotide diversity (*µ*), haplotype number (Nh) and haplotype diversity (Hd). The neutrality test-based Tajima’s *D* was calculated based on segregation sites, using the same software. The haplotype network was constructed based on a median-joining model with 1000 iterations and default parameters using the PopArt software.

## Results

### Sequencing and sequence analysis

Amplification of the 1.2-kb PCR-F fragment was achieved for all seven reference strains and for 16 of the 26 clinical strains. For the remaining samples, two additional PCR reactions for amplification of the PCR-N and PCR-T fragments were performed, followed by sequence assembly. All amplification products derived from the wild samples were obtained by re-amplification, over the first product of PCR-N and PCR-T using the same primer set for each sample.

### Species typing and phylogenetic relationships

**Fig. 1:**
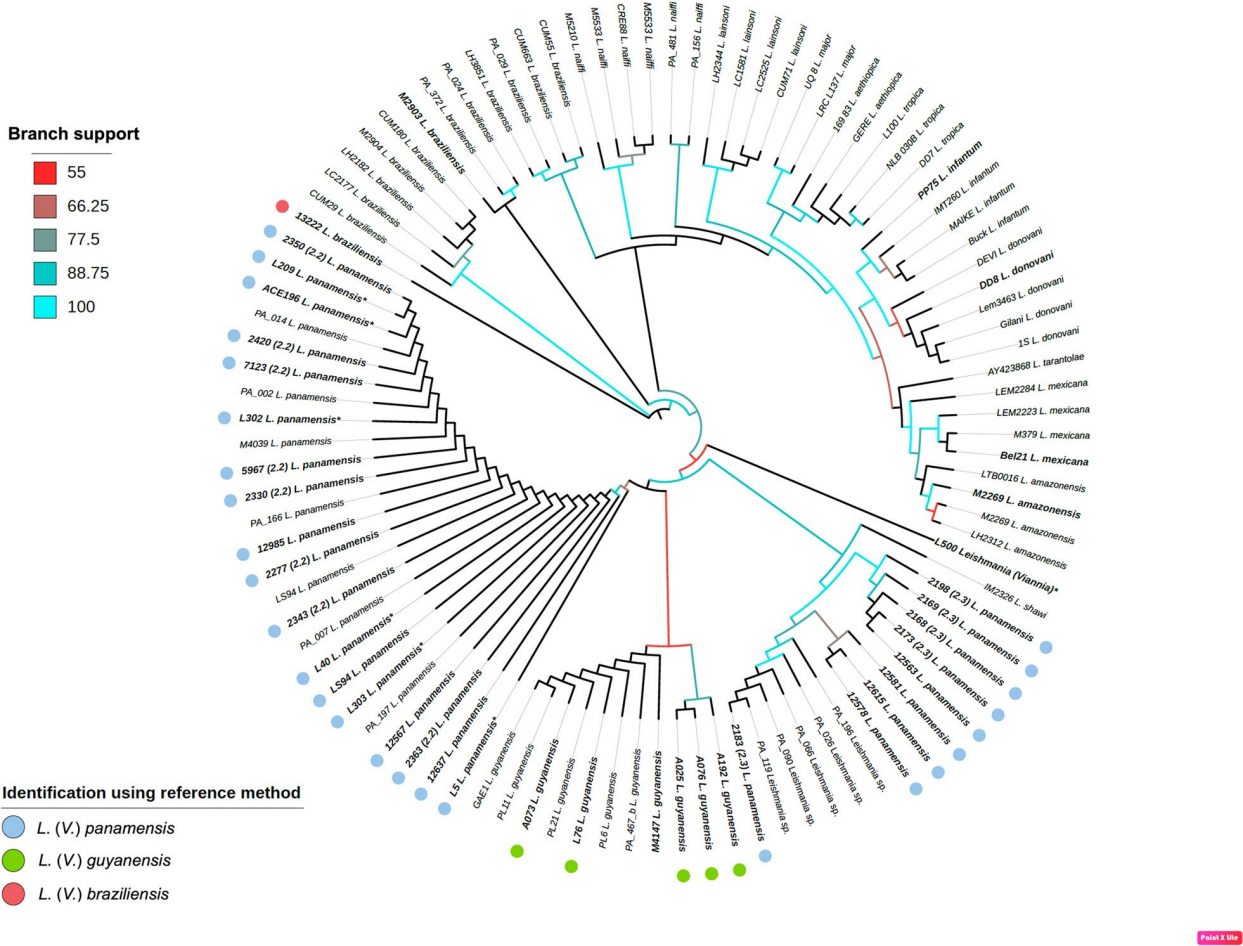
Phylogeny of *Leishmania* spp. from partial nucleotide sequences of the *hsp70* gene. The tree was constructed with IQ-TREE using maximum-likelihood bootstrap support (1000 replicates). The new sequences contributed by this study are indicated in bold. Branch support values are represented by a color gradient. *Leishmania* (*Viannia*) *panamensis* strains are marked with blue dots, *L.* (*Viannia*) *guyanensis* with green dots and *L.* (*Viannia*) *braziliensis* with red dots. Reference sequences are labeled with strain codes and abbreviated species names. Sylvatic strains are marked with an asterisk. Abbreviations: *hsp70*, Heat-shock protein 70,

Using previously published and curated sequences (*n* = 59; Additional file 1: Table S1) in addition to the 41 sequences from this work, we assembled a final alignment of 100 *Leishmania* strains. The whole alignment is shown in Additional file 2: Figure S1, including the consensus sequence (at 50% conservation) and residue conservation that were calculated. Phylogenetic trees were generated using ML and BI (model of substitutions for the ML tree was HKY + F + I and for the BI tree, HKY + I). As expected, the phylogenetic analysis separated the *Viannia* from *Leishmania* clades (Fig. 1). Both trees corroborated the distinctive division between the *Leishmania* and *Viannia* subgenera, as well as the different complexes and species (Additional file 3: Figure S2). Based on *hsp*70 sequences, the identity of all of the clinical isolates analyzed (*n* = 26) was 100% congruent to the species previously defined by monoclonal antibodies or MLEE typing (Table 1). This result was corroborated by the assignment of all seven sequences to the reference strains analyzed (Fig. 1).

Two different clusters of *L.* (*V.*) *panamensis* strains were observed in the phylogenetic trees. In one cluster, nine strains (codes 12581, 12615, 12578, 2183, 12563, 2198, 2168, 2173 and 2169) isolated from patients with CL from the South Pacific coast of Colombia typed as *L.*(*V.*) *panamensis* clustered with a group of reference sequences of *Leishmania* sp. from Panama [41]. Five of those nine strains were also typed as *L.*(*V.) panamensis* zymodeme 2.3 using MLEE (Fig. 1). In the other cluster, 11 *L.*(*V.*) *panamensis* clinical strains (codes 2363, 5967, 2277, 7123, 2420, 2348, 2330, 12567, 2350, 12985 and 12637) grouped with the reference sequences of *L*. (*V.*) *panamensis*, including the strain LS94, in the phylogenetic tree. Of these 11 strains, eight had been previously typed by MLEE as *L.*(*V.*) *panamensis* zymodeme 2.2. *Leishmania* (*V.*) *guyanensis* clinical and reference strains were grouped with lower support values than *L.* (*V.*) *panamensis* and did not display intraspecific separations.

*Hsp70* sequences obtained from vertebrate tissues and insect pools fitted as well as the clinical strains within the whole sequence alignment, with the same resolution at a specific taxon level (Fig. 1). The five sequences of *L.* (*V.*) *panamensis* obtained from *Lutzomyia gomezi* from Córdoba Department clustered with *L.*(*V.*) *panamensis* zymodeme 2.2 clinical strains. The *Leishmania hsp70* sequence obtained from *Oecomys* sp. was also found in this cluster and is congruent with the geographical distribution of *L*. (*V.*) *panamensis* in Colombia. Lastly, the *hsp70* sequence from *Psychodopygus panamensis* (L500) did not group with any specific cluster but was closely related to strains of the *L. guyanensis* complex. This sequence also remained undetermined typed by ITS1 and Mini-exon techniques (data not shown).

### Monophily of* L.* (*V.*) *panamensis* strains

Regarding the phylogenetic topology among *L.* (*V.*) *panamensis* strains, a comparison of marginal likelihood estimates from topologically constrained and unconstrained trees rejected the monophyly of the group based on *hsp70* sequencing. The likelihood value of the constrained tree topology (− 2820.74) was smaller than that of the unconstrained tree topology (− 2811.30). The value of the test statics (BF) was 18.88, indicating very strong evidence in favor of the better model. Therefore, the BF test indicates that the constrained tree topology is not supported, thus bringing into question the monophyly of *L.* (*V.*) *panamensis* based on this marker. Detailed information on the obtained likelihood is given in Additional file 1: Table S2.

### Median-joining network

**Fig. 2:**
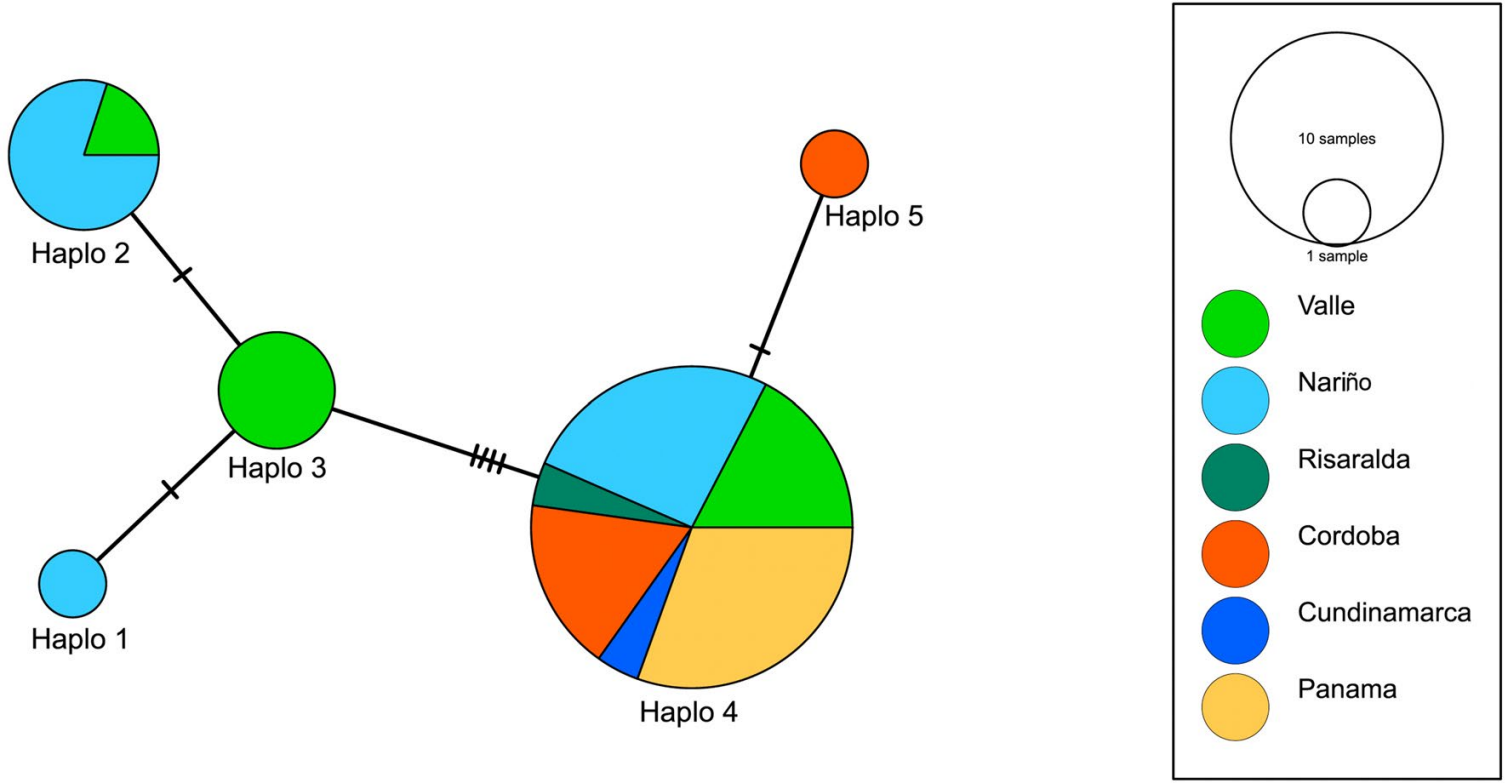
Haplotype of *L.* (*V.*) *panamensis* network based on *hsp70* sequences from this study and reference strains from Panama. Each observed haplotype is indicated by a circle, sized according to its frequency and by number of colored slices according to the localities associated to these. Haplotype relationships are indicated by lines; mutational steps between haplotypes are represented by the number of lines

**Fig. 3:**
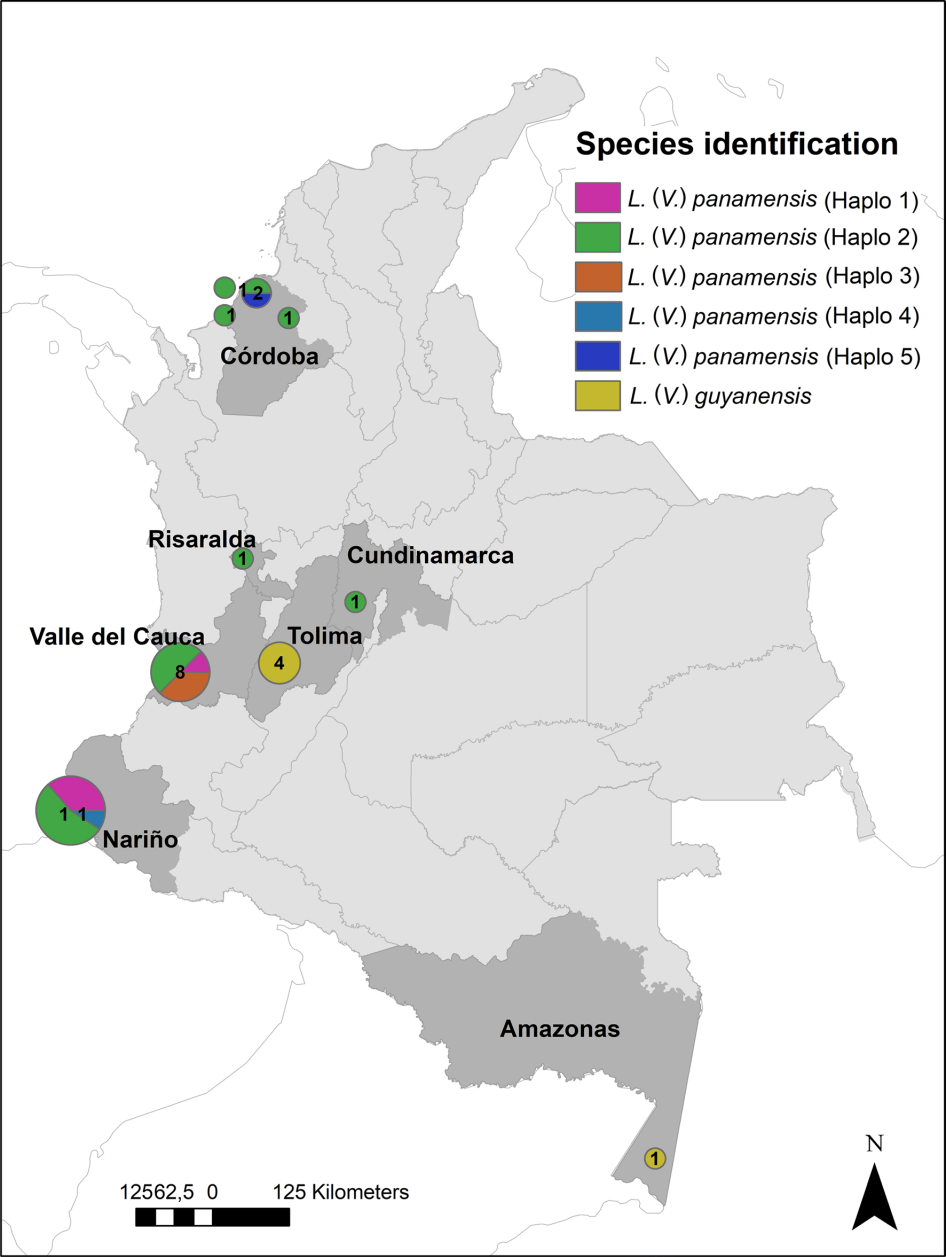
Map of *Leishmania guyanensis* complex presenting the distribution and diversity of the *hsp70* haplotype. The number in each circle corresponds to the total samples analyzed for each location. The haplotypes are color coded distinctively

Seven segregating sites and five parsimony-informative sites were found among the sequences of *L.* (*V.*) *panamensis*, and five haplotypes (H) were defined (Fig. 2). To compare the diversity detected among the strain panel using this genetic region with other published data, we also tested the nucleotide diversity (*µ* = 0.0019) and Tajima’s *D* values (0.8671, *p*[*D* > 0.8671] = 0.4020). Twelve of the *L.* (*V.*) *panamensis* sequences from clinical samples, four of the sequences from vectors and the sequence from the reservoir were grouped in haplotype H2. All of the *hsp70* sequences obtained from *L.* (*V.*) *panamensis* zymodeme 2.2 strains clustered in the H2 haplotype. Haplotypes H1, H3 and H4 corresponded to *L.* (*V.*) *panamensis* sequences generated from *L.* (*V.*) *panamensis* zymodeme 2.3 strains in addition to other clinical strains obtained from patients with CL on the Pacific coast of Colombia (Fig. 3).

## Discussion

Co-circulation of different *Leishmania* species has been reported in Colombia, with *L.* (*V.*) *panamensis* and *L.* (*V.*) *braziliensis* species accounting for most of the human CL cases [42, 43]. We evaluated the performance of *hsp70* gene sequencing for *Leishmania Viannia* genotyping in a panel of clinical strains of *L.* (*V.*) *panamensis* and *L.* (*V.*) *guyanensis,* and for species identification directly from tissue and DNA samples obtained from sand flies and a wild mammal captured in endemic areas of CL transmission in Colombia.

Previous studies in Neotropical countries, such as Ecuador, French Guiana, Brazil, Peru and Panama, have reported the usefulness of *hsp70* sequence analysis for *Leishmania* species identification [41, 37–47]. Our results expand these findings, lending support to *hsp70* sequencing for discriminating closely related species within the *L.* (*V.) guyanensis* complex. Our study provides the research community with an additional number of strains and species from Colombia, thereby enhancing the discriminatory power of sequence-based *Leishmania* species typing, which supports the feasibility of implementing this tool in a wide and heterogeneous geographical range. This characteristic is of great importance in beginning to define a unified method for regional species identification, one of the priorities of local and regional public health programs [48, 49]. By using this approach, we achieved species identification from clinical isolates, mammalian hosts and vectors, highlighting the potential of *hsp70* sequencing in multidisciplinary studies with an ecological and epidemiological scope [49].

In our study, *L*. (*V.*) *panamensis* was over-represented, including clinical strains and DNA samples from a sylvatic mammal and sand flies. Two discrete clusters of *L*. (*V.*) *panamensis* were identified based on the tree topology, BF and haplotype data: one cluster is represented by *L.* (*V.*) *panamensis* strains of zymodeme 2.2 and the other cluster is represented by zymodeme 2.3. MLEE analysis brought into question the distinction between *L.* (*V.*) *panamensis* and *L.* (*V.*) *guyanensis* since data did not indicate distinct monophyletic lines [50]. In addition, the zymodeme 2.3 strains sequenced in this work clustered with high support values together with strains sequenced in Panama by Miranda et al. [41] and identified by the authors as *Leishmania sp.1.* Therefore, a close, if not identical, taxonomic identity is suggested. Interestingly, the authors of previous studies of *Leishmania* (*Viannia*) species circulating in the South Pacific coast region of Colombia concluded that the genetic similarity between the *L*. (*V.*) *guyanensis* and *L.* (*V.*) *panamensis* zymodeme 2.2 was greater than that between the *L.* (*V.*) *panamensis* zymodemes 2.2 and 2.3 [51]. Previous findings [52] demonstrated the distinction of three well-supported clades in a tested panel of *L.* (*V.*) *panamensis* from Colombia and Panama, and a possible geographic distinction between these intraspecific clades. These findings provide evidence of the need for the revision of the taxonomic status of the *L.* (*V.*) *guyanensis* complex.

Among the diverse *L.* (*V.*) *panamensis* strains and parasite populations circulating in Colombia, *L*. (*V.*) *panamensis* zymodeme 2.3 has been associated with higher in vitro profiles of drug tolerance/resistance to pentavalent antimony [53]. HSPs have been previously implicated in antimony resistance in clinical *L*. *donovani* strains [54]. Moreover, parasite exposure to antimony results in differential expression of *hsp70* [55], suggesting that environmental (or clinical) exposure to metals may exert a selection pressure over HSPs, which could result in population genetic diversity and phenotypic associations. Whether any association exists between the drug-susceptible phenotype and the *hsp70* haplotype remains to be determined and corroborated in a larger number of samples.

The implementation of species typing tools based on DNA or complementary DNA (cDNA) targets (such as 18S ribosomal RNA [rRNA] or 7SL RNA) is further supported by its potential usefulness in cases where parasites are difficult to isolate, such as those coming from infected sand flies or asymptomatic mammalian reservoirs. Here, we provide proof-of-concept of the applicability of *hsp70* sequencing for *Leishmania* typing directly from DNA samples collected from naturally infected sand flies and mammals. Of the seven sylvatic samples analyzed, six were classified as *L.* (*V.*) *panamensis,* which is in line with the predominant species circulating in the endemic areas where these samples were collected (the Caribbean and Andean regions). Here w report for the first time a natural infection of *Oecomys* sp. with *L.* (*V.*) *panamensis* in Colombia, expanding the number of known natural interactions [3]. Ocampo et al. [56] registered the natural infection of *Oecomys trinitatus* with *L.* (*Viannia*) spp. in a transmission area of *L.* (*V.*) *guyanensis* in Colombia; however, these authors did not confirm the parasite identity at the species level. The role of sylvatic mammals in the transmission cycles is key since it is known that transmission rates in certain areas are related to the densities of mammals acting as reservoirs [3], which for *L.* (*V.*) *panamensis* has been traditionally linked to the presence of sloths and spiny rats in the transmission areas [57, 58].

One sequence obtained from a sand fly pool of *Psy. panamensis* was ‘undetermined’ since it did not group closely with any other strains but appeared as an outgroup of the* panamensis*/*guyanensis* complex. This is quite interesting, especially as clinical samples collected in nearby areas were typed as *L.* (*V.*) *panamensis* strains. It is important to note here the possible existence of a sampling bias which leads to the parasites that successfully spread in vertebrate hosts with clinical signs, such as dogs, being more frequently captured, which would have the potential consequence of missing novel sylvatic strains [59].

The haplotype and nucleotide diversity found in this study are similar to those previously reported by Patiño et al. [50] and Van der Auwera et al. [60], indicating the existence of intraspecific variability using the *hsp70* locus. Implementing similar species typing methodologies in future work and on broader geographic scales can increase our ability to offer reliable and comparable information to define the current status of *Leishmania* diversity in Colombia and neighboring countries.

CL outbreaks in Colombia have a dissimilar spatial structure [12], and the strains included in this work represent some of these spatial clusters located in endemic transmission areas. We report here a panel of *hsp70* sequences from Colombian strains belonging to five different geographical areas with distinct environmental conditions; five haplotypes represented among them provide confirmatory evidence of genetically different strains co-occurring in close geographical clusters.

## Supplementary Information

**Additional file 1: Table S1. **Reference sequencesof *Leishmania *spp. obtained from GenBank database for phylogeneticanalysis. **Table S2. **Summary of Bayesfactor comparisons for phylogenetic hypotheses tested: monophyly (constrained)and non-monophyly (unconstrained) of the strains typed as *L. *(*V.*)*panamensis*. All the strains previously typed as *L. *(*V.*)*panamensis* by monoclonal antibodies, MLEE and ITS1 sequencing were included.

**Additional file 2: Figure S1.** Phylogenetic tree andmultiple-sequence alignment analysis for the species typing of Leishmania spp. Thealignment was generated using Geneious Prime. The numbers at the branches are confidencevalues (percentage) calculated based on the bootstrap method. Consensussequence (at 50% conservation) and residue conservation were calculated oniTOL.

**Additional file 3: Figure S2. **HSP70gene majority-rule consensus tree inferred from Bayesian inference by usingMrBayes v.3.2. Posterior probability values from the Bayesian analysis fornodes are indicated below or above branches. Sequences belonging to subgenus *Leishmania*and* Viannia* are shown in green and blue color, respectively.

## Data Availability

The nuclear DNA sequences of *Leishmania* spp. obtained in this study were deposited in GenBank database (Table 1).

## Abbreviations

BF: Bayes factor
BI: Bayesian inference
CL: Cutaneous leishmaniasis
Hsp70: Heat-shock protein 70
ITS1: Internal transcribed spacer 1
kDNA: Kinetoplast DNA
ML: Maximum likelihood
MLEE: Multilocus enzyme electrophoresis
RAPD: Random amplified polymorphic DNA
