# Supplementary material for: Sequencing of hsp70 for discernment of species from the Leishmania (Viannia) guyanensis complex from endemic areas in Colombia
Source: Parasit Vectors. 2022 Nov 3;15:406. doi: 10.1186/s13071-022-05438-w (PMC9635106; doi:10.1186/s13071-022-05438-w)
Supplement: Supplementary file 1 — Additional file 1: Table S1. Reference sequencesof Leishmania spp. obtained from GenBank database for phylogeneticanalysis. Table S2. Summary of Bayesfactor comparisons for phylogenetic hypotheses tested: monophyly (constrained)and non-monophyly (unconstrained) of the strains typed as L. (V.)panamensis. All the strains previously typed as L. (V.)panamensis by monoclonal antibodies, MLEE and ITS1 sequencing were included. [file 13071_2022_5438_MOESM1_ESM.docx]

Table S1. Reference sequences of *Leishmania* spp. obtained from GenBank database for phylogenetic analysis.

| **Strain code** | **Species** | **Accession number** | **Reference** |
| --- | --- | --- | --- |
| 169-83 | *L. aethiopica* | FN395020.1 | Fraga et al. [29] |
| GERE | *L. aethiopica* | FN395018.1 | Fraga et al. [29] |
| LTB0016 | *L. amazonensis* | L14605.1 | Bock & Lager, [30] |
| M2269 | *L. amazonensis* | MG029123.1 | Espada et al. [31] |
| CUM180 | *L. braziliensis* | FN395039.1 | Fraga et al. [29] |
| CUM29 | *L. braziliensis* | FN395041.1 | Fraga et al. [29] |
| CUM555 | *L. braziliensis* | FR872760.1 | Odiwuor et al. [38] |
| CUM663 | *L. braziliensis* | FR872761.1 | Odiwuor et al. [38] |
| LC2177 | *L. braziliensis* | FN395042.1 | Fraga et al. [29] |
| LH2182 | *L. braziliensis* | FN395040.1 | Fraga et al. [29] |
| LH3851 | *L. braziliensis* | FR872763.1 | Odiwuor et al. [38] |
| M2904 | *L. braziliensis* | JF449366.1 | Ramirez et al. [32] |
| PA_029 | *L. braziliensis* | KX573945.1 | Miranda et al. [37] |
| PA_029 | *L. braziliensis* | KX573945.1 | Miranda et al. [37] |
| MAIKE | *L. chagasi* | FN395035.1 | Fraga et al. [29] |
| DEVI | *L. donovani* | MN728741.1 | Fernandez-Arevalo et al. [33] |
| LEM3463 | *L. donovani* | MN728759.1 | Fernandez-Arevalo et al. [33] |
| GAE1 | *L. guyanensis* | HF586406.1 | Van der Auwera et al. [28] |
| M4147 | *L. guyanensis* | KX573934.1 | Miranda et al. [37] |
| PA_467 | *L. guyanensis* | KX574011.1 | Miranda et al. [37] |
| PL11 | *L. guyanensis* | MW658405.1 | Van der Auwera et al. [34] |
| PL21 | *L. guyanensis* | MW658386.1 | Van der Auwera et al. [34] |
| PL6 | *L. guyanensis* | MW658403.1 | Van der Auwera et al. [34] |
| BUCK | *L. infantum* | MN728753.1 | Fernandez-Arevalo et al. [33] |
| Gilani | *L. infantum* | MN728756.1 | Fernandez-Arevalo et al. [33] |
| IMT260 | *L. infantum* | FN395032.1 | Fraga et al. [29] |
| CUM71 | *L. lainsoni* | FN395047.1 | Fraga et al. [29] |
| LC1581 | *L. lainsoni* | FN395048.1 | Fraga et al. [29] |
| LC2525 | *L. lainsoni* | FN395050.1 | Fraga et al. [29] |
| LH2344 | *L. lainsoni* | FN395049.2 | Fraga et al. [29] |
| LRC-L137 | *L. major* | FN395023.1 | Fraga et al. [29] |
| UQ_8 | *L. major* | FN395022.1 | Fraga et al. [29] |
| LEM2223 (SOLIS) | *L. mexicana* | HF586401.1 | Van der Auwera et al. [28] |
| LEM2284 (LM) | *L. mexicana* | HF586413.1 | Van der Auwera et al. [28] |
| LH2312 | *L. mexicana* | FN395038.1 | Fraga et al. [29] |
| M379 | *L. mexicana* | EU599091.1 | Fraga et al. [29] |
| CRE88 | *L. naiffi* | HF586373.1 | Van der Auwera et al. [28] |
| M5210 | *L. naiffi* | FN395056.2 | Fraga et al. [29] |
| M5533 | *L. naiffi* | GU071183.1 | da Silva et al. [39] |
| PA_156 | *L. naiffi* | KX573968.1 | Miranda et al. [37] |
| PA_481 | *L. naiffi* | MT469994.1 | Miranda et al. [37] |
| LS94 | *L. panamensis* | EU599094.1 | Montalvo et al. [35] |
| M4039 | *L. panamensis* | FN395055.1 | Fraga et al. [29] |
| PA_002 | *L. panamensis* | KX573937.1 | Miranda et al. [37] |
| PA_007 | *L. panamensis* | KX573939.1 | Miranda et al. [37] |
| PA_014 | *L. panamensis* | KX573942.1 | Miranda et al. [37] |
| PA_166 | *L. panamensis* | KX573971.1 | Miranda et al. [37] |
| PA_197 | *L. panamensis* | KX573975.1 | Miranda et al. [37] |
| IM2326 | *L. shawi* | GU071175.1 | da Silva et al. [39] |
|  | *L. tarentolae* | AY423868.1 | Brochu et al. [36] |
| DD7 | *L. tropica* | FN395025.1 | Fraga et al. [29] |
| NLB_030B | *L. tropica* | FN395026.1 | Fraga et al. [29] |
| PA_024 | *Leishmania sp.* | KX573943.1 | Miranda et al. [37] |
| PA_026 | *Leishmania sp.* | KX573944.1 | Miranda et al. [37] |
| PA_066 | *Leishmania sp.* | KX573946.1 | Miranda et al. [37] |
| PA_090 | *Leishmania sp.* | KX573953.1 | Miranda et al. [37] |
| PA_119 | *Leishmania sp.* | KX573961.1 | Miranda et al. [37] |
| PA_196 | *Leishmania sp.* | MT469987.1 | Miranda et al. [37] |
| PA_372 | *Leishmania sp.* | KX573991.1 | Miranda et al. [37] |

Table S2. Summary of Bayes factor comparisons for phylogenetic hypotheses tested: monophyly (constrained) and non-monophyly (unconstrained) of the strains typed as *L. (V.) panamensis*. All the strains previously typed as *L. (V.) panamensis* by monoclonal antibodies, MLEE and ITS1 sequencing were included.

|  | Constrained | | Unconstrained | |
| --- | --- | --- | --- | --- |
| Run | Arithmetic mean | Harmonic mean | Arithmetic mean | Harmonic mean |
| 1 | -2773.81 | -2820.17 | -2757.33 | -2801.39 |
| 2 | -2766.76 | -2821.11 | -2755.69 | -2811.99 |
| Total | -2767.46 | -2820.74 | -2756.20 | -2811.30 |
