# Supplementary figures and images for: Sequencing of hsp70 for discernment of species from the Leishmania (Viannia) guyanensis complex from endemic areas in Colombia
Source: Parasit Vectors. 2022 Nov 3;15:406. doi: 10.1186/s13071-022-05438-w (PMC9635106; doi:10.1186/s13071-022-05438-w)

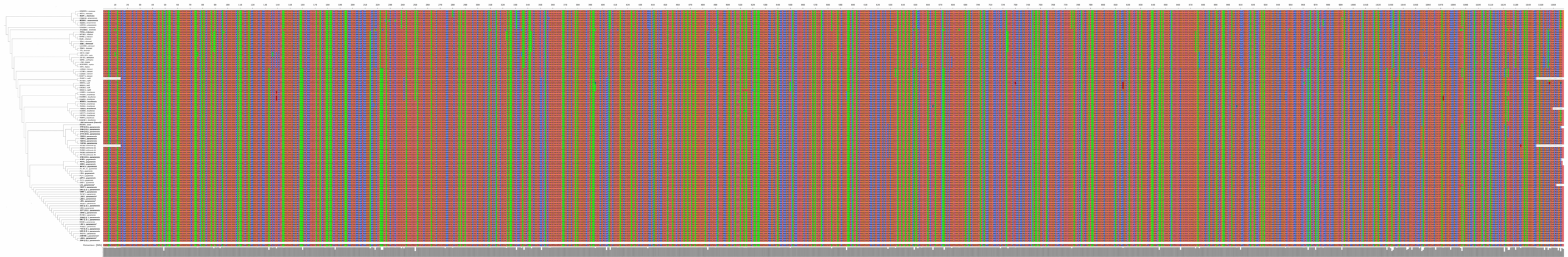

Supplement: Supplementary file 2 — Additional file 2: Figure S1. Phylogenetic tree andmultiple-sequence alignment analysis for the species typing of Leishmania spp. Thealignment was generated using Geneious Prime. The numbers at the branches are confidencevalues (percentage) calculated based on the bootstrap method. Consensussequence (at 50% conservation) and residue conservation were calculated oniTOL. [file 13071_2022_5438_MOESM2_ESM.jpeg]

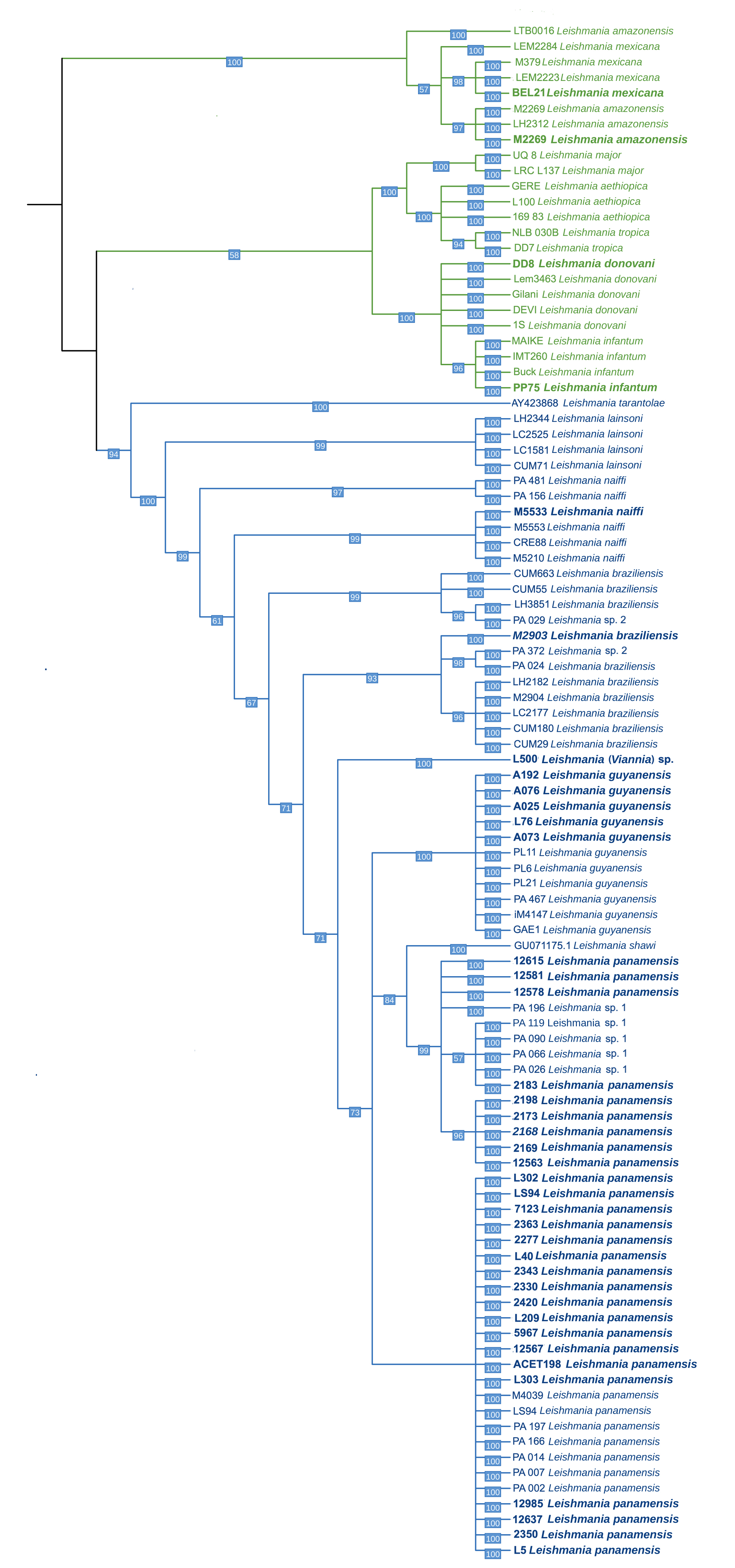

Supplement: Supplementary file 3 — Additional file 3: Figure S2. HSP70gene majority-rule consensus tree inferred from Bayesian inference by usingMrBayes v.3.2. Posterior probability values from the Bayesian analysis fornodes are indicated below or above branches. Sequences belonging to subgenus Leishmaniaand Viannia are shown in green and blue color, respectively. [file 13071_2022_5438_MOESM3_ESM.jpeg]
